# Community-onset *Klebsiella pneumoniae* pneumonia in Taiwan: clinical features of the disease and associated microbiological characteristics of isolates from pneumonia and nasopharynx

**DOI:** 10.3389/fmicb.2015.00122

**Published:** 2015-02-18

**Authors:** Yi-Tsung Lin, Yu-Ping Wang, Fu-Der Wang, Chang-Phone Fung

**Affiliations:** ^1^Division of Infectious Diseases, Department of Medicine, Taipei Veterans General Hospital Taipei, Taiwan; ^2^School of Medicine, National Yang-Ming University Taipei, Taiwan

**Keywords:** capsular types, *Klebsiella pneumoniae*, MLST, nasopharynx, pneumonia

## Abstract

*Klebsiella pneumoniae* is an important cause of community-onset pneumonia in Asian countries and South Africa. We investigated the clinical characteristics of *K. pneumoniae* causing community-onset pneumonia, and the associated microbiological features between *K. pneumoniae* isolates from pneumonia and those from the nasopharynx in Taiwan. This study was conducted at the Taipei Veterans General Hospital during July, 2012 to February, 2014. The clinical characteristics in patients with community-onset *K. pneumoniae* pneumonia were analyzed. *K. pneumoniae* isolates from the nasopharynx of adults attending otorhinolaryngology outpatient clinics were collected to compare their microbiological features with those from pneumonia. Capsular genotypes, antimicrobial susceptibility, and multilocus sequence type (MLST) were determined among these strains. Ninety-one patients with community-onset *K. pneumoniae* pneumonia were enrolled. We found a high mortality (29.7%) among these patients. Capsular types K1, K2, K5, K20, K54, and K57 accounted for ∼70% of the *K. pneumoniae* isolates causing pneumonia, and ∼70% of all the *K. pneumoniae* strains isolated from the nasopharynx of patients in outpatient clinics. The MLST profiles further demonstrated the genetic relatedness between most pneumonia isolates and those from the nasopharynx. In conclusion, our results show that community-onset pneumonia caused by *K. pneumoniae* was associated with high mortality and could have a reservoir in the nasopharynx. To tackle this high-mortality disease, the distribution of capsular types in the nasopharynx might have implications for future vaccine development.

## INTRODUCTION

*Klebsiella pneumoniae* is recognized 100 years ago as a potential cause of community-acquired pneumonia (CAP). However, over the past few decades, *K. pneumoniae* has been a rare cause of CAP in North America, Europe, and Australia (Shon et al., 2013). By contrast, it is an important cause of lower respiratory tract infection (LRTI) in Asian countries and South Africa and is associated with high mortality (Wang et al., 2005; Yu et al., 2007; Song et al., 2008; Lin et al., 2010a,b). In one prospective surveillance study from eight Asian countries, *Streptococcus pneumoniae* (29.2%) was the most common bacterial pathogen, followed by *K. pneumoniae* (15.4%; Song et al., 2008). In Cambodia, *K. pneumoniae* accounted for 8% of community-acquired LRTI (Rammaert et al., 2012). A previous study in an island in the Indian Ocean 2,500 km east of the South African coast demonstrated that *S. pneumoniae* and *K. pneumoniae* (42 and 22%, respectively) were the most frequent microbial agents in CAP patients admitted to intensive care units (Paganin et al., 2004).

In Western countries, although *K. pneumoniae* is a rare cause of CAP, one recent study highlighted that CAP caused by *K. pneumoniae* was associated with increased risk of cardiovascular events following this infection (Griffin et al., 2013). Recently, the emergence of fatal bacteremic CAP due to *K. pneumoniae* was reported in France (Decre et al., 2011; Rafat et al., 2013). This suggests that the re-emergence of severe *K. pneumoniae* pulmonary infections in Western countries cannot be overlooked.

*Klebsiella pneumoniae*, especially the virulent capsular types K1 and K2, has emerged as the major cause of community-acquired liver abscess in the past three decades in East Asia, especially Taiwan (Fung et al., 2002; Fang et al., 2004; Lin et al., 2013). However, the clinical features and microbiological characteristics of *K. pneumoniae* causing community-onset pneumonia have rarely been addressed (Lin et al., 2010a,b). The pathogenesis of *K. pneumoniae* causing community-onset pneumonia is unknown and nasopharyngeal colonization may precede pneumonia.

In the current study, we investigated the clinical features of community-onset pneumonia caused by *K. pneumoniae* in our hospital. We further compared the microbiological characteristics between *K. pneumoniae* strains from the nasopharynx in adult patients attending otorhinolaryngology outpatient clinics and those from community-onset pneumonia.

## MATERIALS AND METHODS

### STUDY DESIGN AND PATIENTS

A retrospective study was carried out in the Taipei Veterans General Hospital from July, 2012 to February, 2014. All consecutive adult patients (aged > 20 years) admitted with community-onset pneumonia due to *K. pneumoniae* were included. Community-onset pneumonia refers to both CAP and healthcare-associated pneumonia (HCAP). Polymicrobial infections were excluded in this study. CAP, HCAP, and hospital-acquired pneumonia were diagnosed based on the criteria previously described (Maruyama et al., 2013). HCAP included patients with pneumonia and any of the following: (1) hospitalization for 2 days during the preceding 90 days; (2) residence in a nursing home or extended care facility; (3) home infusion therapy (including antibiotics); (4) chronic dialysis (including haemodialysis and peritoneal dialysis) during the preceding 30 days; or (5) home wound care. Taiwan is endemic for *K. pneumoniae* liver abscess and cases of liver abscess with septic lung metastasis were also excluded.

We also consecutively collected *K. pneumoniae* isolates from the nasopharyngeal region in adult patients (aged > 20 years) attending otorhinolaryngology outpatient clinics from July, 2013 to February, 2014 to determine their clinical and microbiological characteristics. The main diagnosis of patients attending otorhinolaryngology outpatient clinics during this study period were acute sinusitis, chronic sinusitis, and allergic rhinitis. The protocol was approved by the hospital’s institutional review board.

### DATA COLLECTION AND OUTCOME MEASURES

The following data were collected on admission to hospital: age, sex, comorbid illnesses, clinical symptoms, concomitant bacteremia, and antimicrobial treatment. The Pneumonia Severity Index (PSI) was determined in all patients (Fine et al., 1997). In this observational study, we focused on the following endpoints: 28-day and in-hospital mortality, receiving mechanical ventilation during hospitalization and requiring intensive care.

### MICROBIOLOGICAL EVALUATION

The Vitek 2 automated system (bioMérieux, Marcy l’Etoile, France) was used for bacterial identification and antimicrobial susceptibility testing. The following antimicrobial agents were assayed: ampicillin, cefazolin, cefuroxime, cefoxitin, ceftriaxone, ceftazidime, cefepime, piperacillin–tazobactam, ertapenem, imipenem, amikacin, gentamicin, ciprofloxacin, levofloxacin, trimethoprim–sulfamethoxazole, and tigecycline. To determine the capsular genotypes of *K. pneumoniae*, we undertook *cps* genotyping by the polymerase chain reaction detection of K-serotype-specific alleles at *wzy* loci, including serotypes K1, K2, K5, K20, K54, and K57, as described previously (Fang et al., 2007). Other capsular types were determined by a simple and useful capsular genotyping method for *K. pneumoniae* based on *wzc* sequences as described previously (Pan et al., 2013). *rmpA* (regulator of the mucoid phenotype), a gene known as an extracapsular polysaccharide synthesis regulator, can positively control the mucoid phenotype of *K. pneumoniae.* The presence of *rmpA* was proposed as an important virulence factor (Yu et al., 2006). One recent study showed that plasmid *rmpA* genes could be co-inherited together with the adjacent virulence genes carried by a large plasmid in *K. pneumoniae* (Hsu et al., 2011). Plasmid *rmpA* and *rmpA2* genes were determined as described previously (Hsu et al., 2011).

Multi-locus sequence type (MLST) was performed on all isolates according to the protocol described on the *K. pneumoniae* MLST website (http://www.pasteur.fr/recherche/genopole/PF8/mlst/). MLST results were analyzed using the international *K. pneumoniae* MLST database created in 2005 at the Pasteur Institute in Paris, France (Diancourt et al., 2005).

### STATISTICAL ANALYSIS

The χ^2^ test or Fisher’s exact test were used in the comparison of categorical data, and the Student’s *t*-test or Mann–Whitney *U* test were used for the comparison of continuous variables. Univariate and multivariate logistic regression analyses were performed to predict 28-day mortality. Variables that showed a significant difference (*P* < 0.1) in the univariate analysis were included in the forward likelihood ratio, stepwise multivariate logistic regression model to determine if any of them were independently related to outcome. Analysis was performed using SPSS version 19.0 (SPSS Inc., Chicago, IL, USA) with *P* < 0.05 considered statistically significant.

## RESULTS

### CLINICAL CHARACTERISTICS OF PATIENTS WITH COMMUNITY-ONSET *K. pneumoniae* PNEUMONIA AND RISK FACTORS FOR MORTALITY

During the study period, a total of 191 consecutive patients with monomicrobial *K. pneumoniae* pneumonia were identified. Of these patients, 91 (47.6%) were classified as having community-onset infection, and the remaining 100 (52.6%) were classified as having hospital-acquired infection. Among the 91 patients diagnosed with community-onset *K. pneumoniae* pneumonia, there was a male predominance (*n* = 76) and the mean age was 77.9 ± 11.7 years. CAP accounted for 51.6% (*n* = 47) of the patients. None of the cases had concomitant distant abscesses. The 28-day mortality was 29.7% (27 patients). The overall in-hospital mortality was 34.1% (31 patients). **Table 1** lists the clinical features of CAP and HCAP. HCAP patients had a significantly higher Charlson Comorbidity Index and PSI scores than CAP patients. CAP patients had a higher level of appropriate antibiotic use. Both the 28-day mortality and in-hospital mortality were not different statistically between CAP and HCAP.

**Table 1:** Clinical features of CAP versus HCAP caused by *Klebsiella pneumoniae.*

Characteristic	CAP (*n* = 47)	HCAP (*n* = 44)	*P*-value
Age (year), mean ± SD	76.9 ± 12.2	80.0 ± 11.1	0.387
Male sex	36 (76.6)	40 (90.9)	0.066
Underlying diseases				
Diabetes mellitus	21 (44.7)	21 (47.7)	0.771
Malignancy	13 (27.7)	16 (36.4)	0.373
Neurological disorders	10 (21.3)	15 (34.1)	0.171
Chronic kidney disease stage ≥4	4 (8.5)	9 (20.5)	0.104
Chronic lung diseases	15 (31.9)	10 (22.7)	0.326
Liver cirrhosis	2 (6.7)	1 (3.6)	1.000
Alcoholism	4 (8.5)	3 (6.8)	1.000
Congestive heart failure	8 (17.0)	6 (13.6)	0.655
Charlson Comorbidity Index	2.5 ± 1.9	4.0 ± 2.7	0.003
PSI score	135.3 ± 32.9	153.3 ± 43.7	0.030
PSI, class IV and V	41 (87.2)	41 (93.2)	0.487
PSI, class V	29 (61.7)	31 (70.5)	0.379
Days of hospitalization	23.3 ± 20.2	21.8 ± 17.7	0.695
Initial presentation with septic shock	22 (46.8)	13 (29.5)	0.091
Initial presentation with respiratory failure	23 (48.9)	23 (52.3)	0.750
Requirement of mechanical ventilation during the course	18 (38.3)	21 (47.7)	0.364
Intensive care unit admission	30 (63.8)	26 (59.1)	0.642
Complicated with bacteraemia	12 (25.5)	12 (27.3)	0.851
Appropriate antibiotics	45 (95.7)	35 (79.5)	0.018
Early mortality (deaths ≤48 h)	4 (8.5)	5 (11.4)	0.734
28-day mortality	12 (25.5)	15 (34.1)	0.372
In-hospital mortality	14 (29.8)	17 (38.6)	0.373

Values given as mean ± SD or number of patients (%).

### MICROBIOLOGICAL CHARACTERISTICS OF *K. pneumoniae* FROM PATIENTS WITH COMMUNITY-ONSET PNEUMONIA

**Table 2** shows the distribution of capsular type among *K. pneumoniae* isolates. Capsular types K1, K2, K5, K20, K54, and K57 accounted for 67% (61/91) of all the *K. pneumoniae* isolates. During the same collection period for *K. pneumoniae* isolates from outpatients, the rate of capsular types K1, K2, K5, K20, K54, and K57 was consistent (72%, 31/43). Plasmid *rmpA* and *rmpA2* genes were identified in 65 and 63 isolates, respectively. All but two strains belonging to the six capsular types (K1, K2, K5, K20, K54, and K57) carried the plasmid *rmpA* or *rmpA2* genes. Seventy-one of the 91 (78%) isolates had the wild-type antibiotic susceptibility (susceptible to several classes of antibiotics, except for intrinsic resistance to ampicillin). MLST profiles were further determined in the isolates with capsular types K1, K2, K5, K20, K54, and K57 (**Table 3**).

**Table 2:** Capsular type distribution among *K. pneumoniae* isolates collected from patients with community-onset pneumonia and nasopharynx from outpatients.

Capsular type	CAP isolates No. (*n* = 47)	HCAP isolates No. (*n* = 44)	Nasopharynx from outpatients No. (*n* = 71)
K1	13	9	14
K2	12	9	20
K3	0	1	1
K5	2	1	2
K10	0	1	0
K16	3	0	2
K20	3	2	5
K21	1	0	0
K22	0	1	0
K25	0	0	1
K28	0	1	0
K37	0	1	0
K39	0	0	1
K52	0	0	3
K54	3	2	3
K57	3	2	5
K58	0	1	0
K61	0	1	0
K62	2	2	6
K64	0	3	4
K68	1	0	0
K74	0	1	0
K79	1	1	0
Others^a^	3	5	4

^a^Other capsular types included KN2 (*n* = 3), probable new capsular types (*n* = 4) and non-typeable (*n* = 1).

**Table 3:** MLST profiles among *K. pneumoniae* isolates collected from patients with community-onset pneumonia and nasopharynx from outpatients.

Capsular type and MLST profiles	CAP No. (*n* = 36)	HCAP No. (*n* = 25)	Nasopharynx from outpatients No. (*n* = 49)
K1	13	9	14
ST23	13	9	13
ST260	0	0	1
K2	12	9	20
ST65	4	3	8
ST86	5	2	9
ST373	1	2	2
ST375	2	2	1
K5	2	1	2
ST76	1	0	0
ST660	1	0	0
ST705	0	1	0
ST1049	0	0	2
K20	3	2	5
ST268	2	1	2
ST420	1	1	0
ST1544	0	0	3
K54	3	2	3
ST29	3	1	2
ST714	0	0	1
ST889	0	1	0
K57	3	2	5
ST218	1	0	2
ST592	2	2	3

Values given as number of patients.

### CAPSULAR TYPE OF *K. pneumoniae* ISOLATED FROM NASOPHARYNX IN OUTPATIENTS

Seventy-one isolates from the nasopharyngeal region collected consecutively in otorhinolaryngology outpatient clinics were available for capsular genotyping. Of the 71 patients (45 were male), the mean age was 55.3 ± 15.6 years. Most patients (57/71, 80.2%) were healthy and no underlying diseases were documented. Diabetes mellitus (6/14, 42.9%) and malignancy (6/14, 42.9%) were the most common underlying diseases among the other 14 patients. Most of them did not receive any antibiotic in the visit. *K. pneumoniae* accounted for 11.5% (39/340) of all bacteria isolated from nasopharyngeal region from November, 2013 to February, 2014.

The detailed capsular types are shown in **Table 2**. Capsular types K1, K2, K5, K20, K54, and K57 accounted for ∼70% (*n* = 49) of all the *K. pneumoniae* isolates. Plasmid *rmpA* and *rmpA2* genes were identified in 55 and 52 isolates, respectively. All *K. pneumoniae* isolates that belonged to these six capsular types carried plasmid *rmpA* or *rmpA2* genes. Sixty-eight *K. pneumoniae* isolates showed the wild-type antibiotic susceptibility. Among the other three isolates, one showed an extended-spectrum β-lactamase phenotype, and the other two showed resistance to fluoroquinolones. The detailed MLST profiles in the capsular types K1, K2, K5, K20, K54, and K57 isolates are shown in **Table 3**. ST23, ST65, ST86, ST373, ST375, ST705, ST268, ST29, ST218, and ST592 were found in isolates from both pneumonia and the nasopharynx.

### COMPARISON OF MICROBIOLOGICAL CHARACTERISTICS AMONG *K. pneumoniae* ISOLATES COLLECTED FROM PATIENTS WITH COMMUNITY-ONSET PNEUMONIA AND OUTPATIENTS

Regarding the microbiological characteristics (**Table 4**), the proportion of capsular type K1, K2, K5, K20, K54, and K57, wild-type antibiotic susceptibility and presence of plasmid *rmpA/A2* were significantly higher in isolates from CAP than those from HCAP. In other words, 8.5% of CAP, 36.4% of HCAP, and 4.2% of outpatients isolates showed multidrug resistance. The characteristics were similar between isolates from CAP and outpatients. However, the proportion of wild-type antibiotic susceptibility and presence of plasmid *rmpA/A2* were significantly higher in isolates from outpatients than those from HCAP.

**Table 4:** Comparison of microbiological characteristics among *K. pneumoniae* isolates collected from patients with community-onset pneumonia and nasopharynx from outpatients.

Characteristic	CAP No. (*n* = 47)	HCAP No. (*n* = 44)	Nasopharynx from outpatients No. (*n* = 71)	*P*-value:CAP vs. HCAP	*P*-value: CAP vs outpatient	*P*-value: HCAP vs outpatient
Capsular type K1	13 (27.7)	9 (20.5)	14 (19.7)	0.422	0.315	0.924
Capsular type K2	12 (25.5)	9 (20.5)	20 (28.2)	0.566	0.752	0.355
Capsular type K1 and K2	25 (53.2)	18 (40.9)	34 (47.9)	0.241	0.573	0.465
Capsular type K1, K2, K5, K20, K54, and K57	36 (76.6)	25 (56.8)	49 (69.0)	0.045	0.369	0.184
Presence of plasmid *rmpA*	40 (85.1)	25 (56.8)	55 (77.5)	0.003	0.305	0.019
Presence of plasmid *rmpA2*	39 (83.0)	24 (54.5)	52 (73.2)	0.003	0.233	0.069
Wild-type antibiotic susceptibility	43 (91.5)	28 (63.6)	68 (95.8)	0.001	0.335	<0.001

Values given as number of patients (%).

### RISK FACTORS FOR MORTALITY

Univariate analyses of the factors associated with 28-day mortality are shown in **Table 5**. The microbiological characteristics of *K. pneumoniae* were not associated with mortality. The logistic regression model showed that malignancy [odds ratio (OR) 4.08, 95% confidence interval (CI) 1.32–12.66, *P* = 0.015) and PSI scores (OR 1.017, 95% CI 1.001–1.034, *P* = 0.042) were independent risk factors for 28-day mortality. Initial presentation with respiratory failure (OR 3.38, 95% CI 0.99–11.44, *P* = 0.051) showed borderline significance in predicting mortality.

**Table 5:** Factors associated with 28-day mortality in adult patients with community-acquired pneumonia (CAP) caused by *K. pneumoniae.*

	Univariate analysis		
Characteristic	Survival (*n* = 64)	Death (*n* = 27)	*P*-value
Age, mean years ± SD	77.8 ± 12.5	80.0 ± 9.8	0.964
Male sex	53 (82.8)	23 (85.2)	0.781
Underlying diseases			
Diabetes mellitus	30 (46.9)	12 (44.4)	0.832
Malignancy	15 (23.4)	14 (51.9)	0.010
Neurological disorders	18 (28.1)	7 (25.9)	0.830
Chronic kidney disease stage ≥ 4	9 (14.1)	4 (14.8)	0.925
Chronic lung diseases	19 (29.7)	6 (22.2)	0.468
Liver cirrhosis	1 (1.6)	2 (7.4)	0.195
Alcoholism	5 (7.8)	2 (7.4)	0.947
Congestive heart failure	11 (17.2)	3 (11.1)	0.466
Charlson comorbidity index	2.8 ± 2.0	4.3 ± 3.0	0.015
Healthcare associated pneumonia	29 (45.3)	15 (55.6)	0.373
Pneumonia Severity Index (PSI), score	134.1 ± 35.0	167.4 ± 39.8	0.001
Initial presentation with septic shock	20 (31.3)	15 (55.6)	0.032
Initial presentation with respiratory failure	26 (40.6)	20 (74.1)	0.005
Intensive care unit admission	35 (54.7)	21 (77.8)	0.043
Complicated with bacteremia	9 (14.1)	3 (11.1)	0.704
Appropriate antibiotics	57 (89.7)	23 (85.2)	0.606
Capsular type K1 and K2	31 (48.4)	12 (44.4)	0.727
Capsular type K1, K2, K5, K20, K54, and K57	43 (67.2)	18 (66.7)	0.977
Presence of plasmid *rmpA*	47 (73.4)	18 (66.7)	0.514
Presence of plasmid *rmpA2*	46 (71.9)	17 (63.0)	0.402
Wild-type antibiotic susceptibility	51 (79.7)	20 (74.1)	0.556

Values given as mean ± SD or number of patients (%). ICU, intensive care unit.

## DISCUSSION

The present study demonstrated the clinical features of community-onset *K. pneumoniae* pneumonia as well as the distribution of capsular types and MLST of *K. pneumoniae* in a medical center in Taiwan. We found a high mortality (29.7%) among these patients. Capsular types K1, K2, K5, K20, K54, and K57 accounted for ∼70% of all the *K. pneumoniae* isolates from patients with pneumonia, and ∼70% of *K. pneumoniae* isolates from the nasopharynx in adults attending otorhinolaryngology outpatient clinics. The MLST profiles further demonstrated several clones common in both pneumonia isolates and those from the nasopharynx.

Only one study has investigated a series of *K. pneumoniae* LRTIs and documented the high fatality rate (15/40, 37.5%) in Cambodia (Rammaert et al., 2012). The current study demonstrated community-onset pneumonia with *K. pneumoniae* had high mortality (29.7%) despite being in a developed country with well-equipped healthcare facilities. Generally, the 30-day mortality rate of CAP is 7.3% in Asia (Song et al., 2008). The mortality rate of overall CAP is 8.3% in Taiwan (Lauderdale et al., 2005). The high mortality caused by *K. pneumoniae* suggested that the virulent strains played an important role in pneumonia. Our previous studies showed high mortality in bacteremic CAP caused by *K. pneumoniae* (Lin et al., 2010b). The current study demonstrated that community-onset *K. pneumoniae* pneumonia is a critical problem in the modern era in endemic areas.

The capsule is a major virulence factor of *K. pneumoniae*, and some capsular types are related to the invasive syndromes (Chung et al., 2012; Siu et al., 2012). The distribution of capsular types in each *K. pneumoniae*-related disease could be crucial for disease control and prevention. We determined the capsular types of *K. pneumoniae* from community-onset pneumonia. We found that capsular types K1, K2, K5, K20, K54, and K57 were prevalent in community-onset pneumonia, which have been reported as the major types for liver abscess in Taiwan (Fang et al., 2007). Most of the applications of MLST for *K. pneumoniae* were for drug-resistant strains and only a few studies have applied MLST to virulent strains (capsular type K1 or K2; Siu et al., 2011; Liao et al., 2014). In the current study, we analyzed the MLST among the virulent capsular types (K1, K2, K5, K20, K54, and K57) from pneumonia, and we found several ST types were prevalent in pneumonia isolates. These virulent clones and the associated increased epidemic potential may be associated with the spread in the community in endemic areas. In western countries, investigations about capsular types of *K. pneumoniae* have rarely been reported. One large-scale study conducted in Europe and North America several decades ago has found that K2 represented the most common types (8.9%) among bacteremic isolates (Cryz et al., 1986). This suggest that capsular K2 type *K. pneumoniae* may be the global threat in this invasive disease.

Patients with HCAP had more exposure to healthcare environments or procedures, and the isolates may have the multidrug resistant problems. However, the virulence factors between CAP and HCAP caused by *K. pneumoniae* have never been compared. In this study, HCAP patients had a significantly higher Charlson Comorbidity Index and PSI score than CAP patients had. However, the number of capsular type K1, K2, K5, K20, K54, and K57, and wild-type antibiotic susceptibility strains were more common in CAP than HCAP. Overall, most of the isolates in this study showed wild-type antibiotic susceptibility. Our previous study on community-onset *K. pneumonia* bacteremia also displayed the prevalent wild-type antibiotic susceptibility isolates (Wu et al., 2012). Although the virulent but less drug-resistant isolates were rarely identified in western countries, they were indeed widespread in Taiwan and Asia countries. It is the remarkable features of *K. pneumoniae* isolates in Taiwan. These virulent capsular types are considered to be closely associated with community-acquired invasive disease or pathogenicity, and are rarely identified in nosocomial strains (Turton et al., 2010; Lin et al., 2014). We found that capsular type K1/K2, traditionally known as the most virulent strains of *K. pneumoniae* types, accounted for 40% of cases of HCAP. We cannot ignore the possible risk of progression to nosocomial spread of these invasive strains, which might result in severe therapeutic problems in immunocompromised hosts.

In our previous study, we suggested that the route of entry in liver abscess is from the gastrointestinal tract (Fung et al., 2012; Lin et al., 2013). We also indicated that none of 49 patients with bacteremic CAP caused by *K. pneumoniae* showed development of *K. pneumoniae* liver abscess concomitantly (Lin et al., 2010b). The pathogenesis of pneumonia and liver abscess may be different and the gastrointestinal tract carriage may not be related to pneumonia. Pharyngeal carriage of *K. pneumoniae* was considered to potentially play a role in the pathogenesis of *K. pneumoniae* pneumonia in recent studies from Asia (Farida et al., 2013; Dao et al., 2014). Farida et al. have shown that the carriage rate of *K. pneumoniae* in healthy individuals in Semarang, Indonesia, exceeds that of *S. pneumoniae* in adults (Farida et al., 2013). Almost all *K. pneumoniae* strains have the wild-type antibiotic susceptibility to common antimicrobial agents; similar to those found in CAP patients in Semarang, Indonesia (Farida et al., 2013). In our findings, most patients from outpatient departments were healthy and had no underlying diseases. Therefore, the findings may represent the colonization status and distribution of capsular types in the general population. Consistent with the recent study conducted by Farida et al. (2013), nearly all the strains from the nasopharynx in our study showed wild-type antibiotic susceptibility. The high prevalence of virulent capsular types (K1, K2, K5, K20, K54, and K57) in the community setting may correspond to the isolates from community-onset pneumonia. The MLST profiles further demonstrated the genetic relatedness between pneumonia isolates and those from the nasopharynx. Several ST types, such as ST23, ST65, and ST86, were prevalent in both pneumonia and the nasopharynx. These findings are a first step in elucidating potential pathways leading to community-onset pneumonia with *K. pneumoniae*.

Our study was limited by its retrospective design. However, our data have important clinical implications because of the lack of capsular types from pneumonia and the nasopharyngeal region reported in the literature. The other limitation was that we could not explore the association between infection and colonization precisely because of the different groups studied. A future prospective study would give us the opportunity to detect colonization and infection by the same *K. pneumoniae* strain and the associated clinical features. Lastly, while most the patients from outpatient departments had no underlying diseases and did not receive antibiotics, they did have upper respiratory tract infections. Collecting nasopharyngeal samples from non-ill healthy adults could be the best method to investigate the nasopharyngeal carriage. Despite this limitation, we give an insight that community-onset pneumonia could have a reservoir in the nasopharynx, rather than being spread from specific sources.

In conclusion, we found a high mortality (29.7%) among community-onset *K. pneumoniae* pneumonia. Capsular types K1, K2, K5, K20, K54, and K57 accounted for ∼70% of all the *K. pneumoniae* isolates from patients with pneumonia, and ∼70% of *K. pneumoniae* isolates from the nasopharynx in adults attending otorhinolaryngology outpatient clinics. These six capsular types and wild-type antibiotic susceptibility strains were more common in CAP than HCAP. The MLST profiles further demonstrated the genetic relatedness between most pneumonia isolates and those from the nasopharynx. The similar microbiological characteristics imply that *K. pneumoniae* isolates in the nasopharynx play a role in the pathogenesis of pneumonia. The distribution of capsular types in the nasopharynx might have implications for future vaccine development.

## AUTHOR CONTRIBUTIONS

YTL contributed to study concept and design, and data analysis and interpretation; YTL and YPW contributed to data acquisition and manuscript drafting; FDW contributed to critical manuscript revision; and CPF contributed to the supervision of this investigation.

## Conflict of Interest Statement

The authors declare that the research was conducted in the absence of any commercial or financial relationships that could be construed as a potential conflict of interest.
